# How Family Living Arrangements and Migration Distances Shape the Settlement Intentions of Rural Migrant Workers in China

**DOI:** 10.3390/ijerph192316308

**Published:** 2022-12-06

**Authors:** Lei Che, Haifeng Du, Xiaoyi Jin, Marcus W. Feldman

**Affiliations:** 1 School of Public Administration, Xi’an University of Architecture and Technology, Xi’an 710055, China; 2 School of Public Policy and Administration, Xi’an Jiaotong University, Xi’an 710049, China; 3 Department of Biology, Stanford University, Stanford, CA 94305, USA

**Keywords:** family living arrangements, settlement intentions, migration distances, migrant children, China

## Abstract

Rural migrant workers and their families will decide the future of China’s urbanization. Using data from the “China Migrants Dynamic Survey and Hundreds of Villages Investigation” carried out in 2018, we examine whether and how family living arrangements and migration distances shape rural migrant workers’ settlement intentions in urban areas. In general, rural migrant workers’ settlement intention is shown to be weak. However, individuals with children are more likely to have a stronger intention to settle permanently in urban areas. Among geographical factors, geospatial distance exerts a negative influence on migrant parents’ settlement intention when the interaction effect of family living arrangements and migration distances is considered. Migrant families are increasingly concentrated in cities near their hometowns with a low entry barrier that allows them to gain access to better amenities. Socio-economic factors, especially disposable income, human resources, and housing conditions, play significant roles in migrant parents’ settlement intention. The age and hometown region of migrant parents are also closely related to their intentions to settle in urban areas. Potential channels for the management of urbanization policy are also explored.

## 1. Introduction

“*Hukou*” is the registration system established in China in 1958, which divides Chinese people into two groups: citizens with urban *hukou* and those with rural *hukou*. It represents a major difference in livelihood, social status, and access to public services [1]. As an industrializing country, China has witnessed rapid urbanization and a substantial increase in internal migration since the early 1980s, with a large number of rural migrant workers changing their means of making a living to jobs in cities where they can make more money and gain the recognition of being an urban resident [2]. Although challenging, settling in cities is essential to achieve this life goal. It is a little-known fact that the rate of change to permanent urban residence (“*changzhu renkou chengzhenhualü*” in Mandarin) has always been higher than the official rate of household registration (“*huji renkou chengzhenhualü*” in Mandarin) by 16%, a phenomenon called “incomplete urbanization” [3]. To respond to calls for the relaxation of controls on *hukou* transfers, the State Council of China planned to transfer the *hukou* of 100 million migrants by 2020. However, there remain huge gaps between the numbers of migrants, eligible applicants, and *hukou* winners [4]. Therefore, accelerating the process of the citizenization of rural migrant workers who have lived in cities for a long time with stable jobs is at the core of improving the process of urbanization [5].

In the past four decades, Chinese families have experienced changes in both size and structure that are embedded in the urbanization process. For a long time, China’s family system maintained strong internal stability, even during long separations, and many social scientists believe that China’s family structure is unlikely to change very quickly [6]. However, the meaning of “family” is culturally charged and susceptible to change during any social transition [7]. Family togetherness, a long and much-valued tradition in Chinese culture, has changed profoundly [8], with rapid urbanization and massive internal migration separating rural parents and children and causing serious tensions in family and marital relationships [9]. In the process of migration, the motivation shifts to self-improvement and family development, and intentions concerning where to make a future home and how to achieve family reunification vary depending on family utilities, life course, and opportunities. Considering the distance involved in family separation, the choice for domestic rural migrants is seen mostly in the pattern of youth migration, parental priority, and parental follow-up [10]. As a result, some scholars have regarded migrants’ choice to separate from their families as a way of maximizing their families’ utilities.

With the change in the economy and rapid social development, the vast gap between urban and rural areas has generated many problems for families. At the end of 2017, among the 287 million internal migrants (the data are from the Report on China’s migrant population development (2017)), the proportion who migrated with their entire families was only 30.35% (34,130 out of 112,455 surveyed in 2017), and family separation became a major problem (Table 1). The state has influenced family decision-making directly through policies regarding the distribution of educational resources according to the student registration management system (the student registration management system uses electronic information technology to record student household registration types, academic performance, physical health, and award or punishment information during education. Like the *hukou* system, it curtails migrant children’s enrolment in inflow city schools by raising the eligibility criteria for admission (Chan 2019)) (“*xueji*” in Mandarin) and the points-based *hukou* system (the points-based *hukou* system is a policy of megacities’ governments that allows non-natives of the city under the legal retirement age who have held a temporary residence permit with the city’s social insurance records and without a criminal record to be eligible to accumulate points towards the city’s *hukou*) (“*jifen luohu*” in Mandarin). This means that the current cost to cities of urbanization is low, not only because many people are transferring from the agricultural sector, providing low-cost labor, but the city has not provided necessary public services to migrants’ family members, such as social insurance, work injury insurance, and children’s right to enter public schools; these are not consistent with the Chinese government’s plan to promote “people-centered urbanization” [11]. Migrant workers and their families are deemed as temporary residents without the right of long-term settlement, and they must return to their hometown when they lose the ability to work. In addition, family dysfunction, reflected in problems with left-behind children and migrant children, has produced behavioral and schooling difficulties [12,13].

Social and economic benefits for urban citizens have not been extended to migrant laborers and their families, whose migration to cities was driven primarily by the urban-rural economic gap. It is hard for migrants to obtain formal urban municipal citizenship [14], and many are now dual-location families [15]. Thus, their migration has not resulted in real citizenization, but in a new pattern of urbanization. Motives for establishing a dual-location family vary from one setting to another. The multi-location livelihood strategies in economic, material, and non-material services are studied by linking the livelihood concept, as an actor-centered approach, with the vulnerability concept. If the social function of the family cannot be realized with dual location, there will be a large number of left-behind children, migrant children, and left-behind women. Using data from the 1995 “China 1% Population Sample Survey”, Liang and Chen [16] found that migrant children are much less likely to be enrolled in school than local children, and that this will have long-term consequences for urban society.

China’s “New Urbanization Development Plan, 2014–2020”, promulgated in 2014, which is concerned with the rural-urban imbalance and insufficient regional development, has produced a series of measures to promote urbanization, with the goal of a 60 percent urbanization rate and high-quality development by 2020. The question of migrant families’ settlement intention in destination cities has not received much attention in the migration literature compared to the extensive empirical research on determinants and effects of population migration from the rural countryside to cities [17,18,19]. In the implementation of the plan, local and nearby urbanization and the development of small and medium-sized cities and towns have played a major role in population absorption and have eliminated some of the social problems brought about by long-distance and cross-regional migration. The rural population follows the pattern of “going out of agricultural production but not leaving the hometown, reuniting, relocating to a town, and obtaining town citizenship” to realize the goal of a better life [20]. A growing body of literature has begun to focus on the determinants of migrant families’ settlement intention [21,22]. Yang [23] shows that different factors play significant roles in individual versus family migration, and highlights the importance of distinguishing between these in studies of industrializing countries. A similar finding is reported by Fan et al. [24], who focused on family migration, arguing that it is not necessarily a prelude to permanent settlement. Migrants work and live in cities, but they are not part of the urban class, nor are their children [25]. In short, migrants go beyond the superficial one-dimensional narrative and should be considered as floating families in different types of cities.

A relationship between migrant families and settlement intention has been shown in some empirical studies [26]. However, previous studies have usually overlooked that settlement intention may differ between solo migration and family migration. Migration or settlement decisions may be complicated when children are involved and geospatial factors are considered. Migrant parents may be concerned with their consumption and income, as well as the well-being and utility of their children, when deciding whether to settle permanently at their destinations. In the context of the new Chinese urbanization and dual household registration, the proportion of couple migration and whole-family migration among all migrants has increased considerably. This provides an opportunity to examine whether and how rural migrant workers’ families life together is associated with their intention to settle in the city. In this paper, we incorporate the migration structure and flow distance, as well as effects of other family members’ follow-up, on migrants’ behavior under different flow patterns. Do experiences of the rural migrant’s family in the city continue to be relevant when deciding to reunite and live an urban life? What factors influence migrant families’ reunification and settlement decisions? What is the status of family migration? This article analyzes the stability of settlement intentions of rural migrants as a function of migration distance and family structure.

We first develop a conceptual framework by reviewing the related literature on how potential factors in settlement intention are related to different family migration patterns. In Table 1, the “China Migrants Dynamic” survey data from 2012 to 2017 were used to examine the trend in family migration patterns. Second, we examine the influence of family reunion patterns and geospatial distance on settlement intentions, assuming that migrants with different family patterns of migration are bound to achieve utility equilibrium. The results show that the proportion of couple migration among all migrants has increased and rural migrant workers tend to migrate to bigger cities in eastern areas of China, where they can receive higher income. In the migration decision for rural migrant workers, the priority is the maximization of economic benefits, so called “economic rationale”, which results in more than 30% (34,659 out of 112,455) choosing to migrate alone (Table 1). The *hukou* system and invisible system barriers are causing separation in agricultural migrant families. In the long experience of migration, rural migrant workers’ families have been forced to adapt to family separation. The driving force of many family decisions is not towards reuniting, but towards separation; in this regard, the current migrant policy is anti-family. Third, considering the Chinese *hukou* system, and the fact that inequality in household registration policies produces a tendency to work in first-tier cities, but settle down in smaller ones, we explore the interaction between migration type and migration distance in settlement decisions.

## 2. Trends in Internal Family Migration in China

In developed countries, age-specific urbanization has gradually leveled with increased economic development [27]. When accompanying family members live together in the city, especially in the later stage of the migration, these reunited families tend to be more stable. The pattern of children’s follow-up often reflects the strength of family resilience, representing the critical “relay” stage in the current citizenization process, and analysis of children’s follow-up patterns can produce a model of family citizenization under different types of urbanization. This is of great practical significance for understanding the dynamics of the population’s citizenship and the effects of laws that govern urban and rural population flow.

Many rural migrant workers have left their spouses and children in their hometowns instead of bringing them to the destination cities. Table 1 shows that the fraction of family migrants in the total sample decreased dramatically from 48.91% in 2012 to 30.35% in 2017. The fraction of long-distance family migrants among family migrants also decreased from 82.39% in 2012 to 79.04% in 2017 (Column 13 in Table 1). The proportion of migrant couples (i.e., their children are at boarding school or taken care of by elders) increased from 21.77% in 2012 to 36.80% in 2017. These patterns exhibit two characteristics, partly due to the new type of urbanization and discrimination against migrants in the urban labor market. First, solo migrants are more likely to be “far away from their hometown”; that is, they usually migrate alone, leaving their spouses and children (if any) behind, and geospatial distance from their families can be enormous, which makes reunions difficult. The geographic distance and cultural distance between origin and destination are negatively related to the intention to settle in destination cities, so migrants often commute between their hometown and urban areas once a year during the Chinese spring festival to reunite with their families [28]. Second, the proportion of “nearby” migrants in “father or mother migrate” families increased from 15.48% in 2012 to 23.96% in 2017 (Column 11 in Table 1). Given their age, lack of skills, and a series of livelihood development-related restrictions, most rural migrant workers take dangerous and low-paying jobs in informal employment sectors, and usually cannot benefit from the social security or housing subsidies available to urban residents. However, *hukou* policies and education systems in nearby small and medium towns are more friendly than in their hometowns, which provides them an opportunity to decide whether and how to achieve family unification at lower costs.

## 3. Literature Review and Framework

### 3.1. Migration Decision-Making in Rural Migrant Worker Families

Research on the logic and economic rationality of settlement intentions has been a major focus of migration studies [29,30,31,32]. Many demographers believe that economic factors influence population migration and are inseparable from human migration behavior. Crozet [33] used gravitational theory to analyze population movements from 1980 to 1990, finding that the population tends to flow to areas with higher market potential and lower price indices. Xu and Wu [34] found that the extent of migration is inextricably linked to the amount of migration benefits. Citizenization is a decision made after a comprehensive assessment of whether the city has provided a stable job and the migrant has adapted to urban life. From an individual perspective, family members’ high-cost constraints may drive rural migrant workers to abandon “carrying their families” and choose solo migration [35]. Since there is a high proportion of family-based mobility (Table 1), the traditional rational economic paradigm that children move with their migrant parents brings challenges. However, children following their parents will inevitably lead to citizenization.

Under the new economics of labor migration, individual rural migrant workers no longer determine livelihood decisions, and the family is regarded as the main entity that pursues expected income maximization and household risk minimization. Children have always played an essential role in Chinese families, and an increasing number of rural migrant workers choose to bring their children or spouses to meet their emotional needs, even if it increases the cost of migration. On the other hand, China’s urban and rural areas have substantial differences in social security and education. The public services enjoyed by urban residents far exceed those in rural areas both in quality and quantity. Children’s education has always been a priority for the Chinese family, and the educational opportunities in destination cities have also increased the desire for family migration. From the perspective of labor needs and to avoid the risks to production and income, or to obtain scarce resources such as human capital, rural families may eventually maximize their expected return by sending one or more family members to other labor markets [20]. An important feature of population migration is the strengthening trend of family migration. It is unfair to interpret family separation only as a “win-win” for both individuals and their families [36]. Family migration is the migration of the rural couple, who work in agriculture and migrate with their children. At present, China’s family-based flow patterns are diverse, with semi-family flows and family flows coexisting; the proportion of semi-family flows is higher. Therefore, migrant families can be divided into four modes of mobility based on the core family: (1) family migration; (2) couple (husband and wife) migrate and separate from children; (3) husband or wife with children; (4) husband and wife move separately, and children stay with another party.

The explosion of rural migrant workers results from the connection between the Chinese land system (China’s dual-track land tenure system parallels that of the rural–urban dual structure. Whereas urban land belongs to the state, rural land is controlled by rural collectives, namely villages. Members of rural collectives are entitled to land rights for two purposes, “contract land” (*Chengbaodi* in mandarin) and “housing land (*Zhaijidi* in mandarin)”. In general, members of rural collectives allocate and divide the rural benefits. Villagers who change their rural *hukou* to urban *hukou* or transfer their hukou to another location will normally lose such benefits. Giving up rural *hukou* without appropriate compensation for rural migrant workers may result in a loss of income and no place to live) and the household registration system, which has been criticized by academics [37]. Due to the rapid development of the “Reform and Opening Up” policy, more cheap labor is needed in order to develop the market economy. In the 1980s, farmers migrated and moved to cities to make up for the gap between urban and rural incomes. In the past ten years, China’s household registration system policy has appeared to be looser, but the restriction on freedom of migration has not changed substantially. In large coastal cities of the southeast and provincial capital city, the system is still strict, and it is difficult for rural migrant workers and their families to obtain fair and just social services [4,38]. Difficulties in obtaining equal pay and development opportunities have limited the mobility of rural migrant workers’ families to a certain extent. However, the household registration system is not the only cause of restricted flow among rural migrant workers’ families.

Emphasis on the household registration status of rural migrant workers as the root cause of their inferior position in urban society has mostly obscured other institutional factors, the most important of which is the dual-track land tenure system. At present, the land’s social security function still exists, but the land can only bring meager direct benefits to rural migrant workers. The land initially exists as an asset, but because it cannot be converted into a source of funds for settlement in the city, it has become an institutional factor that slows peoples’ free movement [39]. Incomplete rural property rights will reduce migration, and improved rights of secure possession will increase migration. Rather than saying that farmers have a strong dependence on the land, it is more accurate to say that farmers are not willing to lose land immediately under the premise of unfair land rights transactions [14]. To promote agricultural modernization, the state vigorously promotes direct income support, production support, and price support for farmers and agriculture. However, low agricultural income is the essence of rural collective land ownership because farmers do not have the power and freedom to dispose of land. This has prompted rural migrant workers to informally manage land for relatives, who usually have very low labor productivity.

At present, there is a gap between urban and rural compulsory education in China that cannot be ignored. This is mainly due to differences in school funding, teacher quality, and school facilities. To receive adequate educational resources, children often follow rural migrant workers and study in the destination cities. However, educational urbanization and population urbanization are currently not coordinated or synchronized. The role of population urbanization in promoting education urbanization is affected by the urban–rural dual system. This has led to an educational exclusion-type urbanization that is based on the household registration system and excludes the rural school-age population. Montgomery [40] believes that rapid urbanization has continued to gather more rural-*hukou* school-age children in cities and towns, and has concentrated high-quality educational resources in urban areas, thus increasing the rural-urban inequality in education and limiting the development of rural education. In the long run, this will weaken the effectiveness and fairness of rural education resources, since most rural-*hukou* school-age children are “pendulum migrants” like their parents. There are decreasing numbers of children in rural areas, and teaching resources there are becoming worse, and even rural high schools are subject to institutional discrimination. In Chinese education, tens of thousands of junior high school graduates must choose vocational high schools to continue their education because local governments adjust the ratio of enrollment in secondary vocational schools to that in general high schools [41].

Split households are common among rural–urban migrants in less developed economies’ and constitute a temporary strategy that provides some flexibility for family livelihood [21]. Although the existing literature has done much to capture the rural-urban migrants’ family structure and explain why they choose to split or reunite [20], research on migrants’ settlement decision across time and geography remains limited. These studies have mostly used surveys of migrants in cities, which makes it difficult to analyze settlement intention as a whole unit including members in rural and urban families [42]. Therefore, the impact of family members’ living arrangement and migration distance on settlement intention is yet to be fully understood. The present study contributes to this body of literature in two ways. First, the existing literature describes settlement intention of urban-rural migrants mostly as a strategy to maximize family income and as a decision primarily influenced by economic factors [24]. However, settlement arrangements of split families result from more sophisticated decisions. The pursuit of happiness and stability of rural migrant workers’ families is included as a mechanism influencing families’ settlement intention by utility incentive, which complements the theoretical discussion on the important role of economic and institutional changes. Based on an empirical analysis of multiple determinants of settlement, the present study goes beyond the economic sphere and provides a nuanced understanding of the complexity of family migration and household arrangements. Second, migrant families tend to circulate between their home villages and host cities. Living arrangements of rural-urban migrant families shift over time and across space [21,43]. This study establishes an analytical framework by not only demonstrating the validity and relevance of the family migration pattern and migration distance to settlement intention for rural–urban migrant families, but also discusses the heterogeneity among generations and regions. In addition, building on conventional studies that focus on family migration in cities [7,21,26], this paper also suggests that more attention should be paid to female labor participation and child development in rural China.

### 3.2. Conceptual Framework

Does every working individual in a city like that city? Does the city as a commodity satisfy the desires of families working there? Studies show that the willingness to move to the city as a family generates utility incentives, such as improved economic benefits, social security, and welfare provided by urban labor; urban life investment with urban life identity; stability and happiness with urban life due to the end of family division. Rural migrant workers who are parents bring their children to live on construction sites, which satisfies their pursuit of family integrity, thereby promoting subjective welfare. Simultaneously, family migration has increased the relative deprivation of vulnerable individuals in the agricultural transfer population during the process of urbanization, which has further strengthened their tendency to stay in the city, their sense of identity as citizens, and the generation of citizenship. Finally, in terms of educational returns, family-based mobility makes the needs of children’s education more urgent [10], because parents invest more time and energy in their children’s learning and development. Children accompanied by parents improve their cognitive ability, which should promote the generation of citizenship. From this, we draw our first research hypotheses:

**Hypothesis** **1.** *Higher family utilities represented by more family members living in the city will result in an increase in settlement intentions.*

**Hypothesis** **1a.** *Compared to solo migrants, rural migrant workers who live with their children in the city are more likely to make a settlement decision.*

**Hypothesis** **1b.** *Compared to solo migrants, rural migrant workers who live with their spouses are more likely to make a settlement decision.*

**Hypothesis** **1c.** *Compared to solo migrants, rural migrant workers who live with their nuclear families are more likely to make a settlement decision.*

The choice of workers’ destination is based on the migrants’ geographical flow, which helps explain the impact of geography on citizenization. As the distance between the outflow and inflow areas increases, the proportion of population movement will decrease. According to Chen and Wang [44], working distance has a significant negative impact on the family migration of rural migrants, and the marginal effect of economic incentives on rural migrants’ urban settlement decreases with the distance to their hometown. Distance exerts a negative influence on rural migrant workers’ settlement intention, whereas population size does not matter at either origin or destination [36]. Instead, local mobility has brought new opportunities for re-urbanization in their hometown to rural migrant workers and their families. Qi, Deng and Fu [45] (2019) believe that the inter-county floating population is more willing and able to stay permanently in cities and towns, and this will become the primary mode of future urbanization in China. Comparing the income from migration to that from remaining in place, moderate mobility over a broader range can meet the requirements of higher income for agricultural migrants and their desire to become citizens, which is conducive to the progress of citizenization.

**Figure 1:**
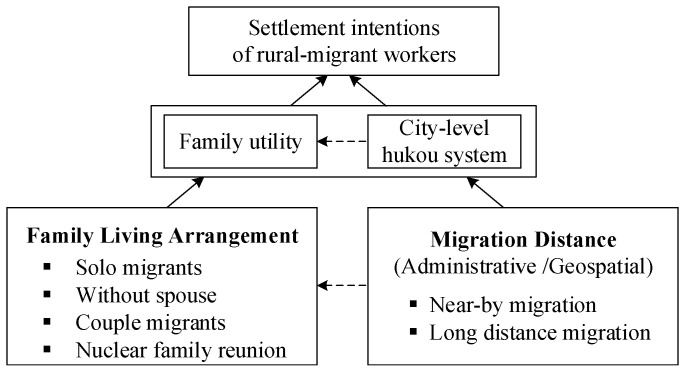
The study’s conceptual framework.

Children’s, spouses’, and parents’ migration may also be affected by the migration distance. The *hukou* and *xueji* registration system in sending cities, which is different from the hometown registration policy, restricts access by rural migrant workers and their families to urban public services, such as getting into a public school, taking the college entrance examination, or using the city’s health services. These significantly increase the separation of families (especially for children and elders) and impede family reunification. The “invisible wall” means that sending cities’ public service systems inevitably raise the barriers to urban admissions and access to public services, especially for inter-provincial migration [46]. In megacities, in the era of the points system for households [47], preferential policies for specific groups are standard for setting entry enrollment barriers for migrant children. Both of these are based on parental conditions for “survival of the fittest” such as education status, technical post title, family planning, investments, and taxes, which make enrollment difficult for children in an ordinary migrant family. The residential segregation of migrants results in the segregation of migrant children’s education and public services. Early studies of rural migrant workers noticed that they live in concentrated areas: “villages in the city” with marginalized and isolated living arrangements [48]. This has negative consequences for migrants’ children, who must enroll in migrants’ schools where “inferior students” are concentrated [49], and for migrants’ family members when they need access to the medical insurance and social pension systems intended for urban residents [50,51]. There are clear effects of flow distance and family migration on settlement behavior. Therefore, we can posit the second hypothesis and conceptual framework (see Figure 1) as follows:

**Hypothesis** **2.** *For the city-level hukou system, nearby migration families can obtain higher utility and enjoy better amenities that will enhance rural migrant workers’ settlement intention.*

## 4. Data and Empirical Strategy

### 4.1. Data and Preliminary Analysis

The data are from a sample survey, “Hundreds of Villages and Towns Investigation” (*Baicun* and *Baizhen*), conducted in 2018 in ten central and west regions with urbanization levels below 60% by the research group on rural migrants from the School of Public Policy and Administration of Xi’an Jiaotong University. The survey involved simple random sampling and was carried out with the assistance of college students from Xi’an Jiaotong University, Henan Agricultural University, Shanxi Normal University, Northwest A&F University, Hunan Normal University, and Huazhong University of Science and Technology in their hometowns during the winter break. The survey sample’s geographical distribution covers western and central areas of China, and, therefore, represents the main outflow areas for the migrant families. In the questionnaire, individuals were defined as rural migrant workers if they were aged 18–45, had left a rural *hukou* location, and lived in the city for more than six months. Although no sampling frame was available, all industries that employed rural migrants were included. The gender, age, marital status, and education of rural migrants were uniformly distributed. The total sample size was 5219, and 4239 families completed the questionnaire. The respondents answered questions concerning their livelihood status and the migration experience of their families. In the sample, 33.61% were first-generation rural migrants, namely, those born in 1979 or before, whereas 66.39% were second-generation, namely, those born in 1980 or after; 78.17% thought their health status was “excellent”; 68.47% had secondary school or technical secondary school education; 42.44% moved to a nearby city; and only 21.30% constituted family migration.

### 4.2. Variables

The desire to become a permanent resident of the destination city and to change one’s *hukou* status are two often used ways to define the dependent variable, according to the current study. When migrants move to a city, a question they need to consider first is whether to stay in this city. This is a decision that almost every migrant must consider. Changing *hukou* is not a requirement, but it is a necessary step for migrants in China to become fully urban citizens and have access to public services and welfare benefits in their new localities. It is also a contract between migrants and the rural local government to give up the benefits attached to the rural *hukou*. The two different definitions are often used in empirical studies. The main purpose of this article is to estimate the rural migrant workers’ settlement intention to permanently settle down in the host city. The dependent variable in the settlement intention equation is a dummy variable, which is 1 if the rural migrant worker responded with “city”, and 0 otherwise.

#### Independent Variables

**Figure 2:**
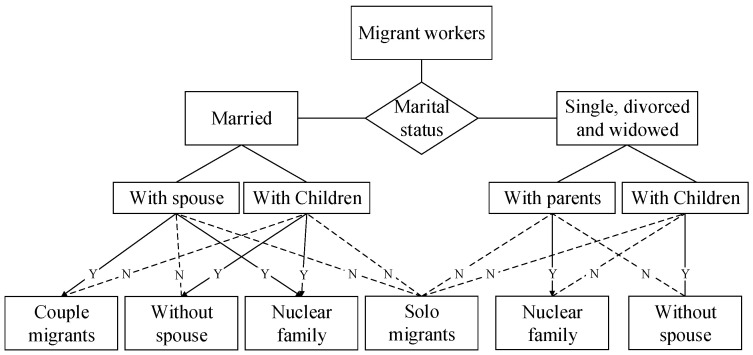
Family living arrangements.

The independent variables are two characteristics of rural migrant workers’ families: with whom they migrate and where they migrate to. Following Fan, Sun, and Zheng [20], we define the “family” as consisting of married rural migrant workers and their unmarried children, or unmarried rural migrant workers and their parents. The “family living arrangements” are identified by one item in the survey questionnaire, “With whom do you live in the city?” “Solo migrants” are those who migrate alone and leave their spouses, children, and parents in their hometown villages. “Without spouse” are those who migrate with their children but leave their spouses in their hometown village. “Couple migrants” are those who migrate with their spouses but leave children in their hometown village. “Nuclear family migrants” are those who are married and migrate with their spouse and children, or those who are single, divorced, or widowed and migrate with their parents (see Figure 2).

Where they migrate to is measured by the question, “In the last six months, where have you mainly been working?” The options include “1. in my home township, 2. in my home county, 3. in my home prefectural-level city, 4. in another prefectural city in my home province, 5. in another province”.

**Figure 3:**
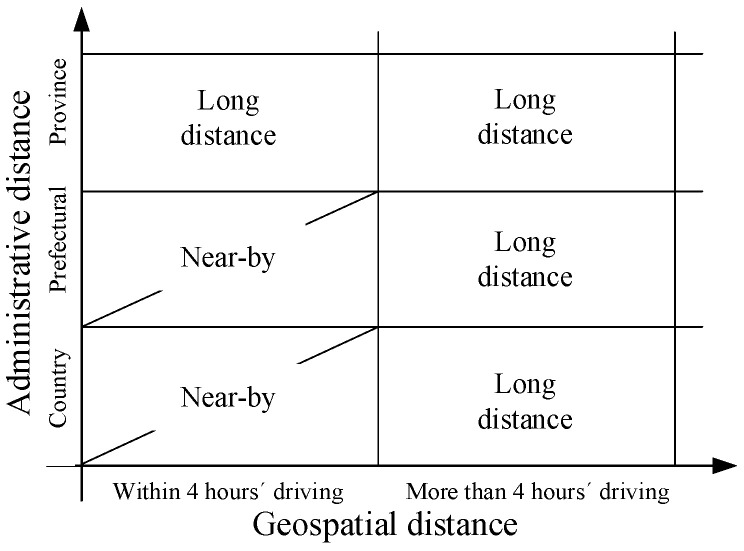
Migration distance.

We calculated the geographical distance according to the latitude and longitude of the inflow and outflow cities. Combining the administrative and geographical dimensions, we propose a migration distance measurement using the county and prefectural level as the basic administrative unit, and four-hours driving as the criterion for geospatial distance. This divides the migration distance into two types: nearby and long-distance migration (see Figure 3).

The control variables include migrant families’ land capital, social capital, human capital, financial capital, and housing assets. Counties of origin are also considered. Land includes the income from farming land and the transfer of farmland management rights. Social capital is measured by whether the migrants belong to a large clan in the countryside and whether they participate in community actives. Human capital has three categories: level of education, health condition, and participation in employment training. Financial capital includes household income, loans or savings amount, and investment status. Housing assets can be divided into property in the hometown village and in the destination city.

Table 2 gives descriptive information on the independent and control variables for migrants. Compared with rural migrant workers who do not have settlement intention, those who migrate alone, with their spouse, or with nuclear family togetherness show a slightly higher willingness to settle down in the city, which shows that the co-living arrangement of family members strengthens settle intentions. The results also shed light on other possible factors affecting settlement intentions (e.g., education, health, work experiences, father’ occupation, employment training, and whether they belong to a large clan in the countryside, etc.). Rural migrant workers who work far from their hometowns show less intention to settle down in the city.

### 4.3. Empirical Model

We now turn to strategies for the analysis of the effect of family migration and the range of migration distance on migrant families’ intention of permanent settlement in a host city. We use a logistic model to perform baseline estimations because the dependent variable is binary:

(1) $${\mathrm{Settlement}\;\mathrm{Intention}}_{i}=\ln(\frac{\pi_{i}}{1-\pi_{i}}{)=\beta }_{0}{+\beta }_{i1}{\mathrm{familymigrant}}_{i}{+\beta }_{i2}{\mathrm{range}}_{i}{+\beta }_{i3}{Χ+\varepsilon }_{i}$$

where *Settlement Intention* is a dummy variable, *familymigrant* is a categorical variable that represents the migration type, *π_i_* is the probability that the settlement intention for case *i* is one, *range* denotes the migration distance between the city and hometown, *X* is a vector that captures the five categories of family livelihood variables: land capital, social capital, human capital, financial capital, and housing assets, and $\varepsilon_{i}$ is the residual term. For convenience, the $\beta_{\mathrm{ij}}$ of the independent variables in the following are all reported as partial regression coefficients and odds ratios.

## 5. Results

### 5.1. Baseline Estimations

We estimate the effect of living arrangements and migration distances on permanent settlement intentions using a traditional logit model and the whole sample. Table 3 presents the results of the logit estimates from Equation (1). Model 1 of Table 3 estimates how family living arrangements are associated with respondents’ permanent settlement intention with control variables included. Model 2 compares the differences between nearby migrants and long-distance migrants with control variables included.

We controlled for a series of family livelihood characteristics (such as income, human capital, and physical capital), which have been shown to have significant impacts on settlement intentions [25,52]. The coefficients for control variables suggest that although there is slight heterogeneity across models 1–4, the willingness to transfer land, belonging to a large clan in the countryside, father’s occupation, education status and health, job training, household income, and having hometown real estate all have significantly positive effects on the intention of staying in the current city. However, having urban real estate, rural land income, and participation in rural social activities have negative effects on the intention to stay in the city, consistent with previous findings [23].

We show the relationship between migration patterns and parents’ permanent settlement intentions relative to the reference group (i.e., solo migrants) in model 3. The odds ratios for migration without a spouse, without children, and nuclear family migrants are 2.438, 1.495, and 1.733, respectively, indicating that the probability that family migrants will settle permanently in the host city is higher than that of solo migrants. Compared with migrant couples, migrant families with children are more likely to stay in the city. Therefore, migrant children have a positive impact on parents’ permanent settlement intentions. Hypothesis 1, 1a, 1b, and 1c cannot be rejected. The results for model 2 show that nearby migration families have a higher probability of settling permanently in the destination city than long-distance migrants (who usually move out of the province). The significance of migration distance suggests that migrants tend to settle down in nearby cities. As shown in model 3, there is a negative effect of geospatial distance on settlement intention, but this is not significant. Compared to model 2, the negative effects of long migration distance become insignificant when we consider family living arrangements, which does not support our second hypothesis. Distance has not dampened rural migrant workers’ willingness to live in their destination cities. Family members’ reunification in the city has a steady positive effect when they reconsider where to live. The findings point to complementarity between family living arrangements and migration distances, which implied that migrants living together with family have a significantly stronger ambition to live permanently in the city.

Model 4 shows interaction effects between family migration type and geospatial distance. In general, compared with solo migrants who migrate to a nearby city, family migration weakens the negative impact of distance on settlement intention. Unlike couple migration that results in the problem of left-behind children, migration with children has a significant positive effect on settlement intentions. If a mother/father migrates to another province with their children, the family is likely to continue to live in the city (the OR of interaction between “without spouse” and “long-distance” is 1.157) because the agricultural production activities, land rights, and care for the aged are provided by their spouses.

### 5.2. Generational Differences

In this section, we explore how the impacts of family migration patterns on settlement intentions vary across generations. Models 5 and 7 in Table 4 show differences in the sensitivity of settlement intentions among rural migrants from different generations to family migration and migration distance, i.e., rural migrant workers born before or in the year 1979, and those born after 1980. In models 6 and 8, we include the main effects and interaction effects of family living arrangements and migration distance.

Model 5 indicates that for the first generation, whole-family migrants have slightly increased willingness to settle down (the OR is 1.155), and for the second generation, moving with their children or family reunification have positive effects (the OR are 1.650 and 1.833, respectively). The influence of migration distance on settlement intention is insignificant across generations (the OR are 0.981 and 1.081, respectively). On the other hand, when family members migrate together, especially with children, they are more likely to settle down in the city, regardless of distance.

For the first generation (model 6), the migration distance’s moderating role is significantly positive between family living arrangements and settlement intentions. Compared with rural migrant workers who migrate to a nearby city alone, when they migrate long distances with their children, their willingness to settle down is increased (the OR is 1.291). For the first generation (model 6), family reunification in a city far from the hometown does not indicate greater intention to settle. The coefficient for second-generation couple migration to a long-distance city is −0.070 (the OR is 0.932, model 8), which suggests that the left-behind children are a major concern in their decision-making. The results indicate that compared to migrating alone to a nearby city, migrating with children to another province has a positive impact on the settlement intentions for the first generation, but migrating without their children to another province has a negative impact on the settlement intentions of the second generation. In other words, in most situations, maximizing utility makes sense in the migrants’ families’ reunification in the city. However, if the second-generation rural migrant workers move to a large city and the parents and children are separated, the overall utility of urban life is reduced in the long term, which supports our second hypothesis. From model 8, the second generation of migrants has a greater demand for livelihood resources, such as employee development opportunities and better children’s education. With increased work experience, migrants with more human and social capital may develop the ambition to chase their “China dream”. However, when they choose distant migration, family separation may significantly decrease their settlement intentions and willingness to become urban citizens.

### 5.3. Regional Heterogeneity

Following the official regional categorization of cities, we divide China into three regions: eastern, central, and western. Models 9, 11, and 13 of Table 5 report the results for the settlement intention model incorporating the effects of family living arrangements and migration distances. In models 9, 11, and 13 of Table 5, the OR for urban reunification of nuclear families indicates whether family reunification affects settlement intention for families from different regions. Models 9 and 11 indicate that among rural migrants from the eastern and central regions, migrating with children or spouses makes it more likely that they will settle in cities and towns, but couple migration does not significantly enhance the willingness to settle. Model 13 indicates that for the western regions, taking any of their family to the destination city significantly increases the settlement intention compared to solo migrants. Therefore, migrant families from the west of China will obtain benefits from reform and urbanization. Compared to solo migrants, the living arrangements of children living in the city with their parents significantly increases the tendency of the family to become urbanized, so the whole family becomes united and lives in the city, which significantly increases their willingness to become citizens.

We report regression results for migration distance’s moderating effect in binary logistic models in models 10, 12, and 14. First, these results provide evidence of a negative effect of distance for the east migration (model 10), which supports our second hypothesis. Specifically, the OR of the interaction term between the without-spouse variable and the long-distance variable is 0.714, indicating that migration across provinces reverses the positive effect of children on rural migrant workers’ settlement intention. Second, for central migration (model 12), the results provide evidence that the family reunification’s effect on settlement intention is reduced by distance, and migrants will choose to migrate with their children and have spouses work in rural production to reduce their urban settlement costs. In model 12, the OR of interaction terms for the without-spouse variable and the long-distance variable is 3.023. Third, the results show that only the long-distance migration of the whole western family can increase their willingness to settle in a big city. The current schooling costs of migrant children in different places are still very high and include residence permits and school choice fees. Since both the content of textbooks and college entrance examinations vary greatly among provinces, migrant children have to go back to their hometowns to finish their schooling in the second year of middle or high school.

Our findings suggest that in terms of migration distance, migrants from the different regions exhibit different migration profiles due to regional imbalances in urban development. The reason could be that migrants from the eastern region can often find jobs with fair pay in small- and medium-sized cities near their hometowns. For families in the eastern region, local migration is an essential way for families to equalize access to public services. Therefore, compared to long-distance migration, short-distance migration significantly increases migrants’ willingness to become citizens. Families from the central and western regions often migrate long distances for greater economic benefits. Although nearby migration has become a recent trend, rural migrant workers in the central and western regions still prefer southeastern coastal cities. The results indicate that although it is always hard to have equal access to public services, unifying the family will maximize the utility of city life.

## 6. Conclusions

In 2013, the People-Oriented New-Style Urbanization Plan outlined in the “National Plan of New Urbanization (2014–2020)”, aimed to address the old strategy’s pressing challenges. It has been in place for more than seven years. Whether the living conditions of millions of rural migrant workers and their families have improved has become the criterion for testing whether this strategy has been successful. Rural migrant workers are not eligible for local public services even though they have moved into the city and participate in the urban labor market. They provide convenience to millions of households, but they are usually separated from their families. There is limited information about, and little attention paid to, China’s rural migrant families’ settlement intention in the context of the different migration patterns and geographical distances. This article attempts to fill this gap by answering whether family living arrangements and migration distance affect rural migrant workers’ settlement intentions. Based on the survey data collected in 2018, our empirical results have three main conclusions.

First, our results highlight that family migration has a significant positive effect on rural migrant workers settling down, but with different marginal effects. The probability that family migrants settle permanently in the host city is higher than that of solo migrants. As the number of migrant families increases, so does the willingness to settle in the city and the frequency of family unification. The results are robust even when different generations and the hometown regional heterogeneity of rural migrant workers are considered. Regardless of their professions and capital status, the second generation is more likely to be influenced by the destination city in terms of lifestyle and means of livelihood and is more willing for the whole family to become citizens and gradually move closer to becoming urban residents [53]. Our results show that families’ urban settlement intention is mainly derived from the utility gained from family unification. The urban social support system increases, but does not determine how family togetherness affects migrants’ settlement intention in the city. To date, the effect of family unification on the stabilization of urban citizenization has not been taken seriously, mainly because the proportion of family migrants is still low (in 2017, about 30.35%, see Column 12 in Table 1). Similarly, a large part of the floating population has chosen to live in a separated status, and the urbanization effect of family unification has been largely ignored. The willingness to move and settle down is a major family decision and involves not only who is united with their family, but who is going to separate first from their family. Thus, a policy implication of our findings is that equalizing the investment in urban education and support for non-agricultural jobs may help increase migrant parents’ intention to bring their children to the city, thereby facilitating the citizenization intention of rural migrant workers.

Second, our findings reveal that people prefer to migrate to nearby small cities rather than to large ones. We find that, for the first generation, migrating with children increases the willingness of the migrants to settle down in distant cities. By comparison, for the second generation, the migration pattern of spouses entering the host city reduces long-distance settlement intention. The second generation of migrants strongly demands livelihood resources, such as employment development opportunities and improved family livelihoods. For some younger couples, it is a necessity rather than an option to leave their children behind. Rural migrant workers from the eastern region of China are more likely to migrate over short distances from regions that are mainly non-agricultural or suburban areas, where there are more service sector jobs. More attention should be paid to long-distance migrating families from the western regions of China through changes in policies that govern how cities support them.

Some scholars, however, have disputed such “local urbanization” or “in-site urbanization” in China and claim that it is a digital game played by local governments to meet their development targets, which, in 2014, aimed to grant official urban residency to 100 million more people by 2020 [54]. The biggest difference between nearby urbanization and long-distance migration to cities is likely to be in their consequences for families’ income growth. As a result, the rural population in a small city is unlikely to become a major contributor to the growth of consumer spending [54]. However, we cannot ignore the nearby migration of surplus agricultural labor, which is a critical socio-economic phenomenon both between and within counties and which profoundly affects prospects for county and city development. Return migration is typically seen in industrializing countries such as China, Vietnam, Ethiopia, and Kenya [55,56,57]. In China’s “Migrant Population Development Report 2017”, the size of China’s floating population showed signs of decline, and the proportions of floating population across cities in the same province (relative to total population) increased annually [58]. This does not mean a reversal of the urbanization process. For returnees from the eastern region, the metropolis is no longer their main destination, and living together with their families in small cities or business hubs with more development opportunities is a priority.

Third, from another point of view, the geographical distance from hometown to host city is explicit, whereas living arrangements between family members are implicit. Both have potentially profound implications on the willingness to become a citizen. Our analysis shows the “distance” between rural and urban migrant workers and citizens and the spatial and psychological distance of migrating individuals and their families during the separation. Our results also show that the desire to become an urban citizen is not just about better individual occupations; it is also about family integrity and meeting the family’s utility. These findings reflect that rural migrant workers’ demands have extended from individual needs to family needs and overall development needs. Thus, the reform of household registration should not only focus on the rural migrant workers’ citizenization, but also on the modernization of rural migrant workers’ families. Our findings emphasize the importance and diversity of settlement intentions among migrant families and provide a theoretical basis for enhancing the life utility of migration and preserving the right to development during urbanization. At the same time, our analysis has incorporated the right to development of other members of rural migrant workers’ families into their decisions about citizenization, to reflect the people-oriented purpose of the urbanization policy, and to provide a new perspective for concern about the problems of left-behind children and migrant children in urban and rural areas.

Our study has the following limitations: first, the sample was collected from a typical rural outflow and the survey was organized during the spring festival. An unobserved group, families that did not return to their hometowns, has a more similar lifestyle to that of urban dwellers and may cause our sample to not be representative across the country. Second, there may be more complicating factors than we addressed when considering the *hukou* transfer decision, or considering lineal families with three generations as the rural migrant family. These may be positively related to the family’s ownership of contracted land and a homestead in the hometown that goes beyond the small families range we discussed and might produce a possible bias because the actual impact of land and related policies on urbanization is neglected. Finally, our study used cross-sectional data. As such, we are unable to assess the bidirectional association between family migration and urban settlement intentions. Studies of international migration show that immigrants’ settlement intentions are closely related to bringing their family members together. Therefore, there is a non-negligible endogeneity between the willingness to settle and family migration status. Future research may consider using longitudinal data for migrants’ family migration pattern to sort out the causal direction and associated mechanisms.

## Figures and Tables

**Table 1:** Trends in family migration patterns, 2012–2017.

Year	Sample Size	Left-Behind Children						Migrant Children					
		Solo Migrants			Couple Migrants			without Spouse			Family Migrants		
		Subsample Size	LD (%)	NB (%)	Subsample Size	LD (%)	NB (%)	Subsample Size	LD (%)	NB (%)	Subsample Size	LD (%)	NB (%)
2012	105,263	29,063	98.99	1.01	22,916	85.15	14.85	1800	84.52	15.48	51,484	82.39	17.61
2013	198,795	61,487	97.50	2.50	42,443	85.84	14.16	2863	75.65	24.35	92,022	79.03	20.97
2014	157,536	47,718	99.08	0.92	33,886	88.37	11.63	2111	71.38	28.62	73,821	79.15	20.85
2015	140,922	39,303	97.88	2.12	36,006	83.57	16.43	2396	72.33	27.67	63,218	77.41	22.59
2016	116,136	34,655	99.94	0.06	43,609	84.24	15.76	1893	78.81	21.19	35,979	79.84	20.16
2017	112,455	34,659	99.60	0.40	41,383	83.87	16.13	2283	76.04	23.96	34,130	79.04	20.96

Source: China Migrants Dynamic Survey (CMDS), 2012–2017. Statistics calculated by the authors. Notes: LD represents long-distance migration and NB represents nearby migration. The family migration patterns (LD and NB) divide into working in a town or city close to their home area, usually within the same province (“*shengnei*” in Mandarin), and those working in cities further afield (“*shengwai*” in Mandarin).

**Table 2:** Summary statistics of variables by settlement intention.

Variables	Definition	Whole Sample	No Intention to Settle Down in the City	Intend to Settle Down in the City
		Mean/Frequency	Mean/Frequency	Mean/Frequency
Settlement intentions	0 = no	33.69	-	-
	1 = yes to stay in the city	66.31	-	-
Family living arrangements	0 = solo migrants	18.94	11.27	23.1
	1 = without spouse	22.67	33.47	17.18
	2 = couple migrants	37.08	34.38	38.2
	3 = nuclear family	21.31	20.88	21.52
Migration distance	0 = nearby	42.44	40.83	43.26
	1 = long distance	57.56	59.17	56.74
**Control variables**				
Annual income from farming land	(Ten thousand)	0.74	0.68	0.77
Willingness to transfer land use rights	0 = not	15.38	18.98	13.48
	1 = conditional transfer	34.02	34.28	33.88
	2 = yes	50.60	46.74	52.64
Belong to a large clan in the countryside	0 = no	67.39	68.75	66.68
	1 = yes	32.61	31.25	33.32
Participate in community actives	0 = no	22.17	25.21	20.58
	1 = yes	77.83	74.79	79.42
Father’s occupation	0 = farmer	60.32	69.71	55.60
	1 = non-farmer	39.68	30.29	44.40
Level of education	0 = primary and below	12.00	20.09	7.74
	1 = secondary and technical	68.47	73.42	65.87
	2 = college and above	19.53	6.49	26.39
Health condition	0 = unhealthy	21.83	28.67	18.24
	1 = healthy	78.17	71.33	81.76
Employment training participation	0 = never	43.70	54.47	37.97
	1 = yes	56.30	45.53	62.03
Annual household income	(Ten thousand)	6.58	5.67	7.07
Loan amount	(Ten thousand)	1.86	1.04	2.28
Investment/Saving amount	(Ten thousand)	2.76	2.09	3.11
Property in hometown village	0 = self-built house	57.83	53.81	59.94
	1 = purchased house	42.17	46.19	40.06
Buying a house in city	0 = no	74.63	83.16	70.16
	1 = yes	25.37	16.84	29.84
	Number of Respondents	4239	1428	2811

Notes: The figures are calculated by the authors using “China Migrants Dynamic Survey and Hundreds of Villages Investigation” in 2018.

**Table 3:** Logit regression results.

Variables	Model 1			Model 2			Model 3			Model 4		
	β	SE	OR	β	SE	OR	β	SE	OR	β	SE	OR
Family living arrangements (ref. solo migrants)												
Without spouse	0.851 ***	0.119	2.342				0.891 ***	0.130	2.438	0.687 ***	0.065	1.987
Couple migrants	0.400 ***	0.111	1.492				0.402 ***	0.122	1.495	0.223 ***	0.103	1.250
Nuclear family reunion	0.515 ***	0.125	1.674				0.550 ***	0.136	1.733	0.460 ***	0.108	1.584
Migration distances (ref. nearby)												
Long distance				−0.801 ***	0.082	0.922	−0.076	0.082	0.927	−0.136	0.180	0.873
Interactions (ref. solo migrants × nearby)												
Without spouse × Long distance										0.146 ***	0.033	1.157
Couple migrant × Long distance										0.070	0.265	1.072
Nuclear family reunion × Long distance										0.688 ***	0.266	1.989
**Control variables**												
Annual income from farming land (ten thousand)	0.101	0.112	1.106	−0.146	0.124	0.904	−0.131	0.125	0.878	−0.130	0.125	0.878
Willingness to transfer land use rights (ref. not)												
Conditional transfer	0.129	0.111	1.138	0.153	0.118	1.138	0.140	0.119	1.151	0.141	0.119	1.151
Yes	0.210 ***	0.106	1.234	0.273 ***	0.113	1.234	0.256 ***	0.115	1.292	0.258 ***	0.115	1.294
Belong to a large clan in the countryside (ref. no)												
Yes	0.088	0.079	1.091	0.148 *	0.085	1.091	0.143 *	0.086	1.154	0.144 **	0.086	1.155
Participate in community activities (ref. no)												
Yes	−0.061	0.090	0.941	−0.010	0.096	0.941	−0.034	0.098	0.967	−0.035	0.10	0.966
Father’s occupation (ref. farmer)												
Non-farmer	0.432 ***	0.080	1.540	0.516 ***	0.086	1.540	0.473 ***	0.087	1.604 ***	0.470 ***	0.087	1.601
Level of education (ref. primary and below)												
Secondary and technical school	0.303 ***	0.117	1.354	0.310 ***	0.123	1.354	0.306 ***	0.125	1.357	0.307 ***	0.125	1.359
College and above	1.231 ***	0.126	3.423	1.416 ***	0.133	3.423	1.318 ***	0.136	3.735	1.319 ***	0.136	3.739
Health condition (ref. unhealthy)												
Healthy	0.171 **	0.090	1.187	0.143 **	0.098	1.187	0.111	0.099	1.118	0.110	0.1	1.117
Employment training participation (ref. never)												
Yes	0.281 ***	0.077	1.324	0.319 ***	0.083	1.324	0.295 ***	0.084	1.343	0.296 ***	0.084	1.345
Annual household income (ten thousand)	0.216 ***	0.058	1.241	0.190 ***	0.036	1.241	0.176 ***	0.064	1.193	0.176 ***	0.064	1.192
Loan amount (ten thousand)	0.009	0.007	1.009	0.015 **	0.008	1.009	0.015 **	0.008	1.015	0.015 ***	0.008	1.015
Investment/Savings amount (ten thousand)	0.001	0.004	1.001	0.002	0.005	1.001	0.002	0.005	1.002	0.002	0.005	1.002
Property in hometown village (ref. self-built house)												
Purchased house	0.608 ***	0.097	1.836	0.450 ***	0.102	1.836	0.501 ***	0.104	1.650	0.502 ***	0.104	1.652
Buying a house in city (ref. no)												
Yes	−0.443 ***	0.077	0.642	−0.419 ***	0.083	0.642	−0.416 ***	0.084	0.659	−0.418 ***	0.084	0.658
Constant	−0.325 ***	0.103	0.727	−0.221 ***	0.224	0.722	−0.297 ***	0.227	0.802	−0.196 ***	0.236	0.822
Pseudo R2	0.1206			0.1157			0.1278			0.1299		
Observations	4239			4239			4239			4239		

Note: * *p* < 0.05; ** *p* < 0.01; *** *p* < 0.001.

**Table 4:** Logit regression results for family migrants’ settlement intentions (by generation).

Variables	First Generation (Born before 1979)				Second Generation (Born after 1980)			
	Model 5		Model 6		Model 7		Model 8	
	β	OR	β	OR	β	OR	β	OR
Family living arrangements (ref. solo migrants)								
Without spouse	−0.173	0.841	−0.071	0.932	0.501 ***	1.650	0.350 ***	1.419
Couple migrants	−0.238	0.788	−0.354	0.702	−0.154	0.857	−0.152	0.859
Nuclear family reunion	0.144 **	1.155	0.197 ***	1.218	0.606 ***	1.833	0.485 ***	1.624
Migration distances (ref. nearby)								
Long distance	0.031	1.031	−0.019	0.981	−0.045	0.960	0.078	1.081
Interactions (ref. solo migrants × nearby)								
Without spouse × Long distance			0.255 ***	1.291			0.477	1.611
Couple migrant × Long distance			0.251	1.286			−0.070 ***	0.932
Nuclear family reunion × Long distance			0.110	0.896			0.036	0.965
**Control variables**	Yes		Yes		Yes		Yes	
Constant	−1.013 ***	0.363	0.359 **	1.431	0.581 ***	1.787	1.930 ***	6.889
Pseudo R2	0.1293		0.1494		0.0912		0.0925	
Observations	1425		1425		2814		2814	

Note: * *p* < 0.05; ** *p* < 0.01; *** *p* < 0.001.

**Table 5:** Logit regression results for family migrants’ settlement intentions (by outflowing cities).

	From Eastern City				From Central City				From Western City			
	Model 9		Model 10		Model 11		Model 12		Model 13		Model 14	
	β	OR	β	OR	β	OR	β	OR	β	OR	β	OR
Family living arrangements (ref. solo migrants)												
Without spouse	0.863 ***	2.370	0.836 ***	2.307	0.934 ***	2.545	0.824 ***	2.280	0.714 ***	2.042	1.009 ***	2.743
Couple migrants	0.275	1.317	0.274 **	1.315	0.364	1.439	0.265	1.303	0.579 ***	1.784	0.841 ***	2.319
Nuclear family reunion	0.478 *	1.612	0.478	1.613	0.481 ***	1.617	0.434 **	1.543	0.844 ***	2.325	1.098 ***	2.998
Migration distances (ref. nearby)												
Long distance	−0.010 ***	0.990	−0.059	0.943	−0.023	0.977	0.025	1.025	0.188 ***	1.206	0.119	1.126
Interactions (ref. solo migrants × nearby)												
Without spouse × Long distance			−0.336 ***	0.714			1.106 **	3.023			0.701	2.015
Couple migrant × Long distance			−0.312	0.732			0.759	2.137			0.605	1.831
Nuclear family reunion × Long distance			−0.164	0.849			0.116	1.123			0.675 ***	1.964
**Control variables**	Yes		Yes		Yes		Yes		Yes		Yes	
Constant	0.782 **	2.186	0.135 **	1.145	1.063	2.895	−0.022	0.978	0.300 ***	1.345	−0.996	0.369
Pseudo R2	0.1118		0.1182		0.1201		0.1206		0.2154		0.2170	
Observations	940		940		2422		2422		877		877	

Note: * *p* < 0.05; ** *p* < 0.01; *** *p* < 0.001.

## Data Availability

The raw data supporting the conclusions of this article will be made available by the authors without undue reservation.
